# Unveiling aucubin-mediated inhibition of PANoptosis in lower limb ischemia-reperfusion injury with a near-infrared H_2_O_2_ fluorogenic probe

**DOI:** 10.1016/j.mtbio.2026.102845

**Published:** 2026-01-23

**Authors:** Tang Deng, Zhanli Peng, Jinxi Liang, Qinghui Kan, Jin Peng, Zhihao Zhou, Lin Huang, Heng Liu, Guiyun Jin, Chen Yao

**Affiliations:** a Division of Vascular Surgery, The First Affiliated Hospital, Sun Yat-sen University, Guangzhou, 510800, China; b Key Laboratory of Emergency and Trauma of Ministry of Education, Department of Interventional Radiology and Vascular Surgery, The First Affiliated Hospital of Hainan Medical University, Hainan Medical University, Haikou, 571199, China; c Department of Ophthalmology, The First Affiliated Hospital of Guangxi Medical University, Guangxi, 530021, China

**Keywords:** Hydrogen peroxide, Aucubin, NIRF probe, PANoptosis, Lower limb ischemia/reperfusion injury

## Abstract

Although reactive oxygen species (ROS)-mediated PANoptosis plays a pivotal role in the pathological dysfunction of lower limb ischemia/reperfusion injury (LL-IRI), its exact mechanism remains unclear. Real-time detection of H_2_O_2_ will help to reveal the complicated relationship between ROS, PANoptosis and LL-IRI. In this work, we designed and synthesized a novel near-infrared fluorogenic (NIRF) probe, BFP-H_2_O_2_, which exhibited a pronounced fluorescence-enhanced response to H_2_O_2_ at 650 nm. BFP-H_2_O_2_ was successfully applied to a TAK1i/LPS-induced PANoptosis cell model, revealing up-regulation of H_2_O_2_ during PANoptosis. To the best of our knowledge, there were no suitable chemical tools available for high-throughput screening of anti-PANoptosis compounds from Plantagodepressa Willd (PW). Leveraging BFP-H_2_O_2_, aucubin (AU) from PW was identified for the first time as the most promising anti-PANoptosis active ingredient. Additionally, BFP-H_2_O_2_ enabled visual imaging of the dynamic changes of H_2_O_2_ in the LL-IRI mice model. Particularly importantly, it was confirmed that AU could effectively inhibit H_2_O_2_-mediated oxidative stress and attenuate PANoptosis in gastrocnemius muscle tissues during LL-IRI. This work not only afforded a reliable chemical tool for screening anti-PANoptosis drugs, but also further elucidated the mechanism of PANoptosis and LL-IRI and advanced the development of therapeutic drugs for PANoptosis-related diseases.

## Highlights

• A novel NIR fluorogenic probe BFP-H_2_O_2_ enabled real-time imaging of H_2_O_2_ during PANoptosis.
• Aucubin was screened via BFP-H_2_O_2_ as a potent natural inhibitor of PANoptosis in LL-IRI.
• Aucubin alleviated LL-IRI via reducing oxidative stress and inhibiting PANoptosis.

## Graphical abstract

**Figure:**
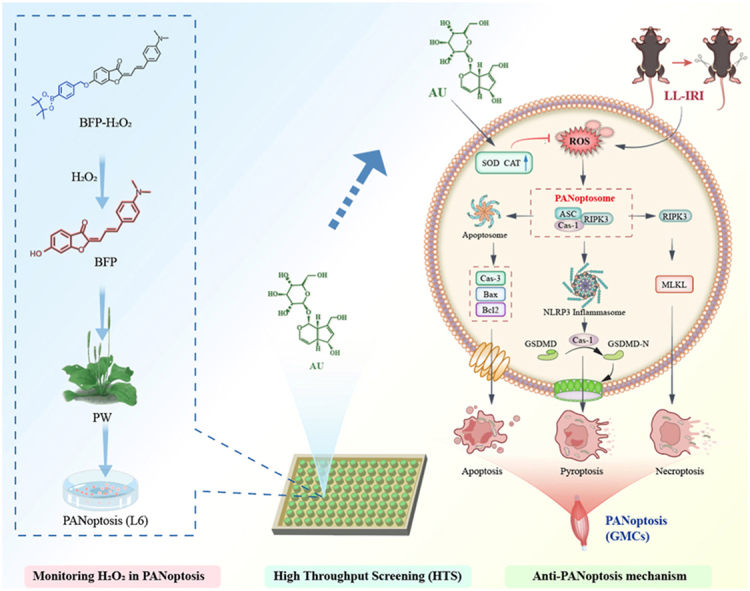

## 1 Introduction

Lower limb ischemia/reperfusion injury (LL-IRI) refers to a systemic immune response triggered following acute or chronic lower limb arterial embolism and the restoration of blood flow through vascular intervention [1]. LL-IRI is primarily driven by oxygen deprivation during ischemia and the subsequent generation of reactive oxygen species (ROS) upon reperfusion, resulting in oxidative stress, inflammatory reactions, and various forms of cell death [2,3]. LL-IRI often brings about necrosis of the limb. Although there is no recommended pharmacological treatment in the relevant guidelines and expert consensus, it is urgent to explore the pathogenesis of LL-IRI and to find non-invasive, cost-effective, and easy-to-administer therapeutic approaches against the background of the current urgent clinical needs [[4], [5], [6], [7]].

Studies show that ROS have a dual role in LL-IRI, not only exerting a vital role in cellular signal transduction, but can also trigger oxidative damage, further leading to apoptosis, necrosis, and other forms of programmed cell death [8]. The concept of PANoptosis, first introduced in 2019, refers to the linked process of multiple forms of cell death, including pyroptosis, apoptosis, and necroptosis, a mechanism associated with the onset and progression of multiple diseases. In recent years, PANoptosis has been recognized as an integrated form of inflammatory programmed cell death characterized by the simultaneous activation of multiple death pathways through shared signaling networks. Increasing evidence indicates that excessive ROS act as upstream triggers that synergistically drive PANoptosis and amplify inflammatory tissue injury. Given the abrupt ROS burst during ischemia-reperfusion, PANoptosis has emerged as a critical pathogenic mechanism underlying acute tissue damage [9,10]. Several ischemia/reperfusion-related studies have shown that excessive ROS is closely coupled with mitochondrial dysfunction, metabolic imbalance, and inflammatory signaling, and ultimately drives tissue damage mediated by PANoptosis [11]. This oxidative stress-related cell death axis has been repeatedly verified in brain, heart, liver, and vascular ischemia/reperfusion models, suggesting that the ROS-PANoptosis pathway plays a crucial role in tissue damage and functional outcomes following reperfusion [[12], [13], [14], [15], [16], [17], [18]]. However, the mechanism of ROS-mediated PANoptosis in LL-IRI is poorly understood, and there is a lack of high-throughput screening platforms to screen relevant anti-PANoptosis drugs [19,20]. Hydrogen peroxide (H_2_O_2_) is one of the most important ROS in the LL-IRI process. H_2_O_2_ not only directly oxidizes proteins, lipids, and DNA, but also acts as a signaling molecule to activate multiple inflammatory pathways and worsen tissue damage [[21], [22], [23]]. Natural products are gradually becoming an attractive source for drug development due to their unique chemical structures and biological activities [24,25]. *Plantago depressa* Willd (PW) is a common Chinese herb, and its dried whole herb is known to possess a variety of pharmacological effects, such as heat-removing and detoxifying, anti-inflammatory, and antioxidant [26]. PW may be a potential drug for the treatment of LL-IRI, implicating its relevance in diseases associated with PANoptosis. To better understand the molecular mechanisms underlying PANoptosis-related diseases and discover effective therapeutic drugs, there is an urgent need for efficient screening tools.

**Scheme 1:**
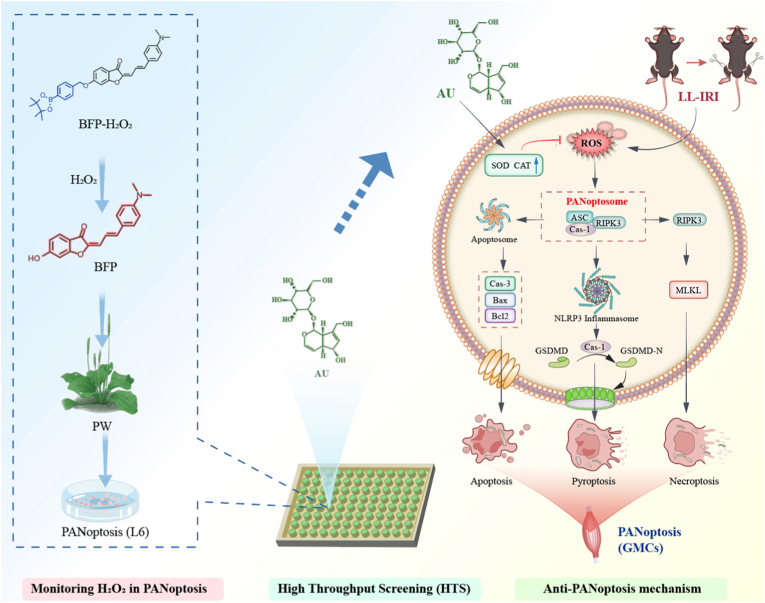
Schematic illustration of the designed H_2_O_2_ NIRF probe for high-throughput screening of anti-PANoptosis natural compounds from PW, and its application in mice model of LL-IRI (by Figdraw).

Fluorescence imaging technology has emerged as an imperative platform for investigating disease mechanisms and screening drugs owing to its high sensitivity, real-time, and non-invasive nature [[27], [28], [29], [30], [31], [32], [33], [34], [35], [36]]. Herein, we reported a novel near-infrared fluorogenic (NIRF) probe, BFP-H_2_O_2_, and successfully achieved real-time monitoring of H_2_O_2_ in the process of LL-IRI (Scheme 1). Further validation results indicated that H_2_O_2_ might serve as a potential biomarker for PANoptosis. The high-throughput screening platform established based on BFP-H_2_O_2_ successfully screened 16 major active ingredients from PW extracts, among which aucubin (AU) exhibited potent antioxidant effects and inhibited LL-IRI-mediated oxidative stress and PANoptosis in gastrocnemius cells.

## 2 Experimental section

### 2.1 Materials and apparatus

The materials, instruments, fluorescence analysis, analyte solution preparation, cytotoxicity, cell culture, and cellular model were listed in the Supporting Information.

### 2.2 Synthesis of (E)-2-((E)-3-(4-(dimethylamino)phenyl)allylidene)-6-((4-(4,4,5,5-tet ramethyl-1,3,2-dioxaborolan-2-yl)benzyl)oxy)benzofuran-3(2H)-one (BFP-H_2_O_2_)

BFP (307 mg, 1.0 mmol), potassium carbonate (276 mg, 2.0 mmol), and 2-(4-(bromomethyl)phenyl)-4,4,5,5-tetramethyl-1,3,2-dioxaborolane (445 mg, 1.5 mmol) were sequentially added to a solution of N, N-dimethyl formamide (10.0 mL). The reaction mixture was heated to 80 °C and stirred for 6 h. The progress of the reaction was monitored by TLC. Upon completion, the solvent was removed under reduced pressure. The crude product was redissolved in dichloromethane and purified by silica gel column chromatography using dichloromethane as the eluent to afford BFP-H_2_O_2_ (183 mg, 35 %). ^1^H NMR (400 MHz, CDCl_3_): 7.85 (d, *J* = 8.0 Hz, 2H), 7.67 (d, *J* = 8.6 Hz, 1H), 7.44 (d, *J* = 8.1 Hz, 4H), 7.07 (dd, *J* = 15.4, 11.5 Hz, 1H), 6.92 (d, *J* = 15.5 Hz, 1H), 6.80–6.77 (m, 2H), 6.74 (d, *J* = 1.6 Hz, 1H), 6.67 (d, *J* = 8.8 Hz, 2H), 5.18 (s, 2H), 3.02 (s, 6H), 1.35 (s, 12H); ^13^C NMR (100 MHz, CDCl_3_): 181.72, 167.11, 165.75, 151.06, 147.12, 141.93, 138.76, 135.17, 131.01, 129.02, 126.53, 125.46, 124.56, 116.42, 116.05, 115.32, 112.23, 111.98, 97.38, 83.89, 70.51, 40.18, 24.84; HRMS *m*/*z*: C_32_H_34_BNO_5_ [M + H]^+^ calcd for 524.2608 found 524.2609.

## 3 Results and discussion

### 3.1 Response of BFP-H_2_O_2_ toward H_2_O_2_

**Fig. 1:**
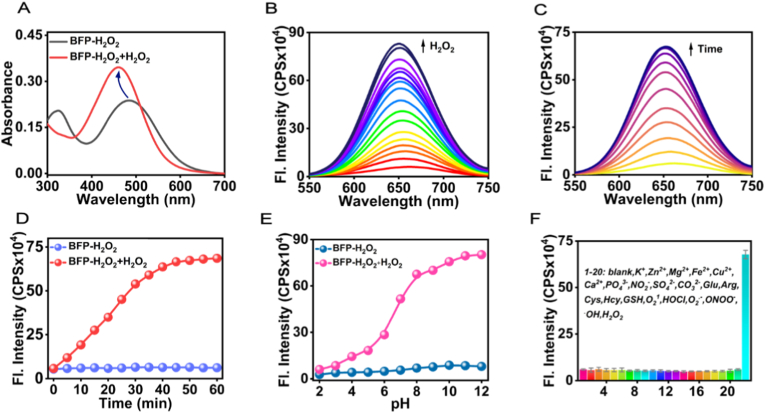
Spectra response of BFP-H_2_O_2_ (10 μM) toward H_2_O_2_ in PBS solution (20 mM containing 30 % EtOH, pH = 7.4). (A) UV-vis absorption changes of BFP-H_2_O_2_ before and after addition of H_2_O_2_ (100 μM). (B) Fluorescence response of BFP-H_2_O_2_ with various concentrations of H_2_O_2_ (0–300 μM). (C) Fluorescence spectra changes of BFP-H_2_O_2_ upon addition of H_2_O_2_ (100 μM). Each spectrum was recorded 5 min after adding H_2_O_2_. λ_ex_ = 490 nm. (D) Time-dependent changes of fluorescence intensities at 650 nm of BFP-H_2_O_2_ in the absence or presence of H_2_O_2_ (100 μM). (E) pH-dependent changes in fluorescence intensity at 650 nm of BFP-H_2_O_2_ with or without H_2_O_2_ (100 μM). (F) Fluorescence response of BFP-H_2_O_2_ to various potential biological analytes (100 μM): 1. blank, 2. K^+^, 3. Zn^2+^, 4. Mg^2+^, 5. Fe^2+^, 6. Cu^2+^, 7. Ca^2+^, 8. PO_4_^3−^, 9. NO_2_^−^, 10. SO_4_^2−^, 11. CO_3_^2−^, 12. Glu, 13. Arg, 14. Cys, 15. Hcy, 16. GSH, 17. ^1^O_2_, 18. HOCl, 19. O_2_^.-^, 20. ONOO^−^, 21. ^.^OH, 22. H_2_O_2_. Data are expressed as the mean ± SD (n = 3).

In this design, BFP-H_2_O_2_ was synthesized via a two-step reaction of the fluorophore BFP with boronic ester derivatives, and its chemical structure was characterized by nuclear magnetic resonance (NMR) and high-resolution mass spectrometry (HRMS) (Figs. S1–S3). BFP featured typical near-infrared fluorescence emission properties and large Stokes shifts, while the boronic ester was a H_2_O_2_-responsive group [[37], [38], [39], [40], [41], [42]]. In the presence of H_2_O_2_, the boronic ester was first oxidized to phenol, followed by a 1,4-elimination reaction, releasing the fluorescent dye BFP, which enabled the detection of H_2_O_2_. The reaction mechanism was verified by HRMS (Fig. S4). To characterize the sensing capability of BFP-H_2_O_2_, UV-vis absorption and fluorescence titration spectra were initially recorded in PBS solution (20 mM containing 30 % EtOH, pH = 7.4). As depicted in Fig. 1A, a strong absorption peak for BFP-H_2_O_2_ was observed at 485 nm, which blue-shifted to 460 nm upon the addition of H_2_O_2_. Fluorescence spectra at varied concentrations of H_2_O_2_ were shown in Fig. 1B. As expected, with the continuous addition of increasing concentrations of H_2_O_2_, the fluorescence intensity of BFP-H_2_O_2_ at 650 nm under the excitation of 490 nm, gradually increased (Fig. S5). At the concentration of 100 μM H_2_O_2_, the fluorescence intensity approached saturation. Notably, a remarkable linear relationship (R^2^ = 0.9935) was established between the fluorescence emission at 650 nm and H_2_O_2_ concentrations ranging from 5 to 80 μM (Fig. S6). Based on the 3σ/slope method (linear fitting equation: y = 6511 x + 143137), the limit of detection (LOD) was determined to be 0.42 μM. As displayed in Fig. 1C and D, the fluorescence intensity of free BFP-H_2_O_2_ at 650 nm remained stable over 60 min, whereas upon the addition of H_2_O_2_, the fluorescence intensity increased significantly and stabilized within 45 min. The pH dependence of BFP-H_2_O_2_ in response to H_2_O_2_ was also evaluated (Fig. 1E). In the absence of H_2_O_2_, the fluorescence intensity of BFP-H_2_O_2_ exhibited only minor changes across a broad pH range from 2.0 to 12.0. Upon the addition of H_2_O_2_, the fluorescence intensity of BFP-H_2_O_2_ increased in the pH range of 3.0–12.0. The fluorescence response of BFP-H_2_O_2_ at pH 7.4 was particularly pronounced, indicating its potential for H_2_O_2_ detection in physiological environments. Selectivity was crucial for the practical application of BFP-H_2_O_2_ in complex biological systems. Therefore, the fluorescence response of BFP-H_2_O_2_ to various potential biological analytes was measured, including metal ions (K^+^, Zn^2+^, Mg^2+^, Fe^2+^, Cu^2+^, Ca^2+^), anions (PO_4_^3−^, NO_2_^−^, SO_4_^2−^, CO_3_^2−^), amino acids (Glu, Arg, Cys, Hcy, GSH), and reactive oxygen species (^1^O_2_, ClO^−^, ONOO^−^, ·OH) (Fig. 1F). Surprisingly, compared to the significant fluorescence enhancement induced by H_2_O_2_, the fluorescence intensity at 650 nm showed negligible changes when treated with other biologically relevant species. These results clearly demonstrated that BFP-H_2_O_2_ was a reliable NIRF probe for monitoring fluctuations of H_2_O_2_ in complex biological environments.

### 3.2 Imaging H_2_O_2_ in live cells

**Fig. 2:**
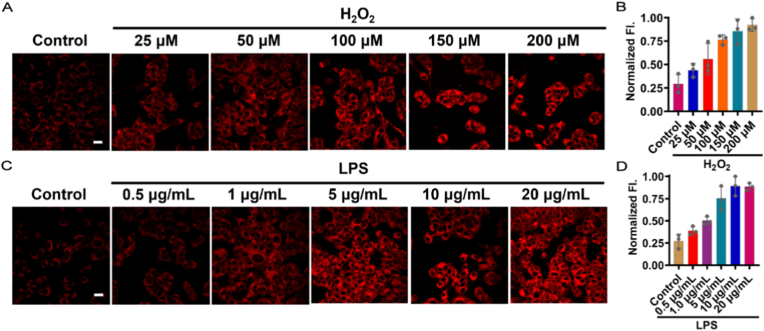
Confocal fluorescence imaging of L-6 cells. (A) Images of cells treated with various concentrations of H_2_O_2_ (0, 25, 50, 100, 150, 200 μM) for 30 min, then incubated with BFP-H_2_O_2_ (10 μM) for 30 min. (B) Images of cells stimulated with LPS (0, 0.5, 1, 5, 10, 20 μg/mL) for 12 h, then incubated with BFP-H_2_O_2_ (10 μM) for 30 min. Scale bar: 20 μm. Values are mean ± SD for n = 3.

Biocompatibility is essential for fluorogenic probes in cellular imaging. To test the cytotoxicity of BFP-H_2_O_2_, L-6 cells were chosen and analyzed using the CCK-8 assay. As depicted in Fig. S7, the cells exposed to BFP-H_2_O_2_ at concentrations ranging from 0 to 50μM for 24h showed a viability over 85 %, indicating that BFP-H_2_O_2_ was well-suited for biological applications. We further evaluated the ability of BFP-H_2_O_2_ to image H_2_O_2_ at the cellular level. Upon treatment with exogenous H_2_O_2_, the intracellular fluorescence intensity increased progressively with rising H_2_O_2_ concentrations (Fig. 2A and B). Likewise, when the cells were stimulated with LPS in the concentration range of 0–20 μg/mL, the fluorescence intensity increased in a concentration-dependent manner, reaching saturation at 10 μg/mL (Fig. 2C and D). In addition, pretreatment with N-acetylcysteine (NAC) markedly decreased the LPS-induced fluorescence enhancement, confirming that the cellular fluorescence response of BFP-H_2_O_2_ was derived from H_2_O_2_ (Fig. S8). These findings illustrated that BFP-H_2_O_2_ enabled real-time dynamic imaging of both exogenous and endogenous H_2_O_2_ levels in live cells.

**Fig. 3:**
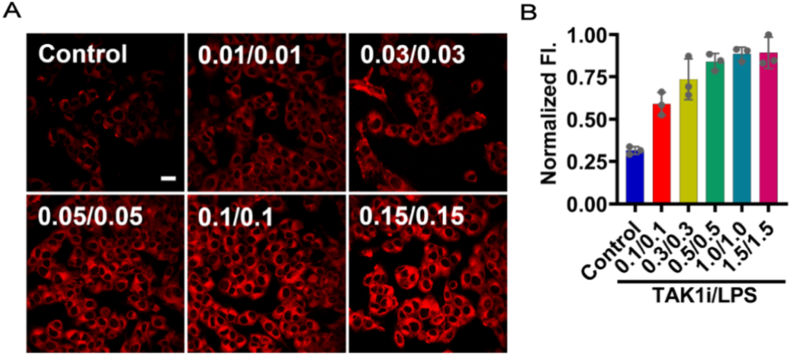
Confocal fluorescence imaging of L-6 cells. (A) Images of cells stimulated with TAK1i/LPS (0.0/0.0, 0.01/0.01, 0.03/0.03, 0.05/0.05, 0.1/0.1, 0.15/0.15 μM/μg/mL**)**, respectively, then incubated with BFP-H_2_O_2_ (10 μM) for 30 min. Scale bar: 20 μm. (B) Normalized fluorescence intensity from images of plane A. Values are mean ± SD for n = 3.

It has been reported that TAK1i/LPS-induced PANoptosis was linked with elevated levels of intracellular oxidative stress [[43], [44], [45]]. H_2_O_2_, as an important mediator of oxidative stress, might play a vital role in the PANoptosis process [46]. To explore the relationship between PANoptosis and H_2_O_2_, L-6 cells were pretreated with TAK1i for 1h and then stimulated with LPS for 3h. After staining BFP-H_2_O_2_ for 30 min, changes in fluorescence signals were monitored (Fig. 3A). Fig. 3B demonstrated that as the concentration of TAK1i/LPS increased, the fluorescence signals were gradually enhanced, plateauing at 0.1 μM/0.1 μg/mL. This indicated that TAK1i/LPS-induced PANoptosis was positively correlated with intracellular H_2_O_2_ level. These results implied that H_2_O_2_ might serve as a potential biomarker for PANoptosis and provide a basis for screening anti**-**PANoptosis drugs.

### 3.3 High-throughput screening of anti-PANoptosis natural compounds from PW

**Fig. 4:**
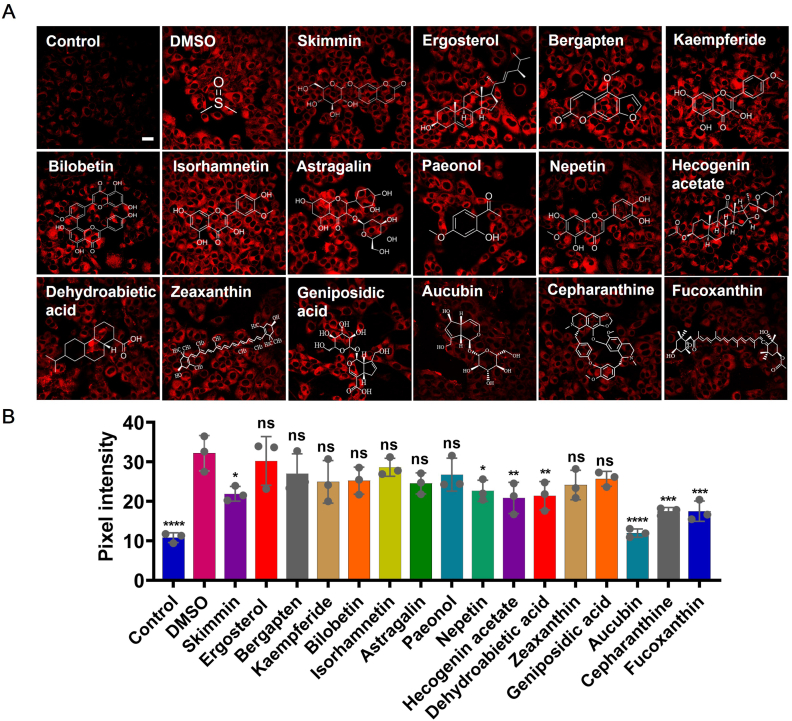
Screening of anti-PANoptosis natural compounds. (A) Images of cells pretreated with 16 major components (100 μM) from PW for 12 h, then stimulated with TAK1i (0.1 μM**)** for 1h and LPS (0.1 μg/mL**)** for another 3h, followed by staining with BFP-H_2_O_2_ (10 μM) for 30 min. (B) Scale bar: 20 μm. (B) Pixel intensity from images of plane (A). Values are mean ± SD for n = 3. ∗P < 0.05, ∗∗P < 0.01, ∗P < 0.05, ∗∗∗P < 0.001, ∗∗∗∗P < 0.0001.

**Fig. 5:**
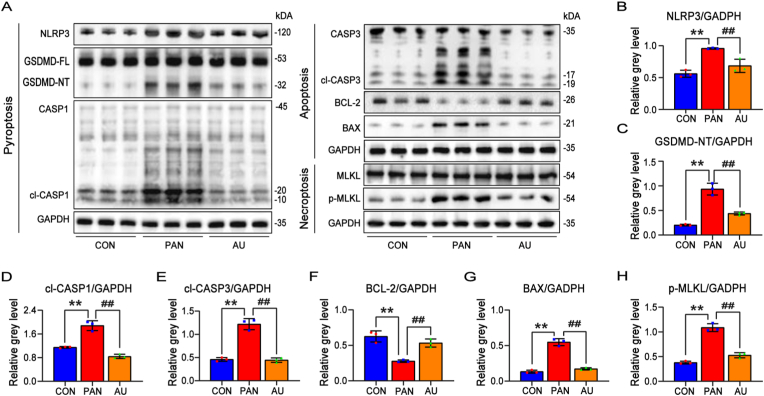
(A) The protein levels of NLRP3、CASP1、GSDMD、Bax、BCL-2、CASP3、pMLKL were detected by Western blot analysis. (B–H) he relative protein expression level of NLRP3、CASP1、GSDMD、Bax、BCL-2、CASP3、pMLKL in (A). Values are mean ± SD for n = 3. ∗P < 0.05, ∗∗P < 0.01, ^#^P < 0.05, ^##^P < 0.01.

**Fig. 6:**
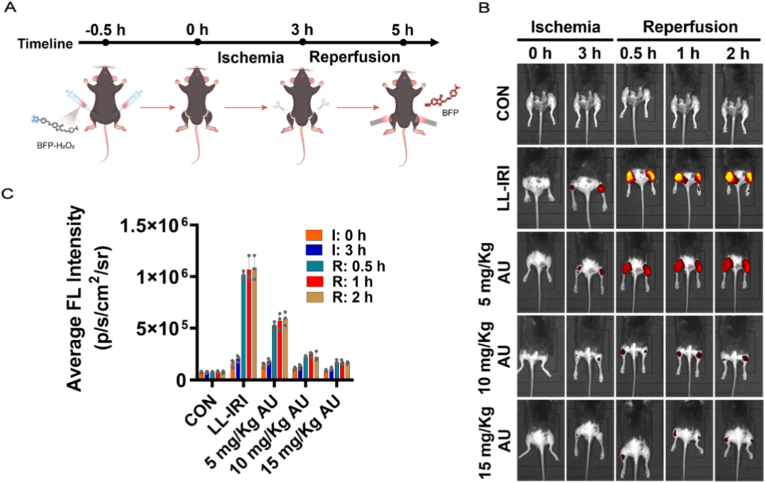
Evaluation of anti-PANoptosis effects of AU in mice model of LL-IRI. (A) Experimental procedure diagram. (B) Fluorescence imaging of H_2_O_2_ in mice model of LL-IRI after in situ injection of BFP-H_2_O_2_ (250 μM, 200 μL). (C)The histogram presents the average FL intensity of mice in panel (a). Values are mean ± SD for n = 3.

PANoptosis is a programmed cell death process orchestrated by multiple cell death pathways, including apoptosis, necroptosis, and pyroptosis [47]. The hallmark of PANoptosis is cellular damage triggered by the accumulation of ROS and mitochondrial dysfunction. Thus, developing natural antioxidants capable of efficiently scavenging ROS and modulating PANoptosis holds significant therapeutic potential. In light of this, we turned our attention to PW, a traditional medicinal herb from the Plantaginaceae family, which is rich in flavonoids, glycosides, triterpenes, and iridoid compounds known for their antioxidant properties. To better understand the bioactive components of PW, high-performance liquid chromatography was employed to determine 16 major compounds (Fig. S9). However, despite their potential, the anti-PANoptosis activity and underlying mechanisms of these compounds have not been systematically investigated. To address this gap, we developed a fluorescence imaging-based screening platform, using intracellular H_2_O_2_ levels in L-6 cells as a key indicator of PANoptosis progression. As shown in Fig. 4A, L-6 cells were pretreated with the 16 isolated compounds for 12 h, followed by co-incubation with TAK1i/LPS to induce PANoptosis. Cells were subsequently stained with BFP-H_2_O_2_ for 30 min before CLSM imaging. Among the tested compounds, AU exhibited the most pronounced anti-PANoptosis activity (Fig. 4B). Further investigation demonstrated that AU exerted a dose-dependent antioxidant effect in TAK1i/LPS-induced PANoptosis (Fig. S10). Consistently, validation experiments in primary gastrocnemius muscle cells (GMCs) showed that AU dose-dependently enhanced cell viability, increased antioxidant enzyme SOD levels, and reduced MDA levels, confirming its cytoprotective and antioxidative effects (Fig. S11).

As depicted in Fig. 5A–H, PANoptosis cells exhibited a marked upregulation of pyroptosis-related proteins NLRP3, GSDMD, cleaved CASP1, apoptosis-related proteins cleaved CASP3, BAX, and necroptosis-related protein phosphorylated MLKL, compared to the control group (CON). Additionally, apoptosis-related protein BCL-2 was downregulated. Notably, AU effectively attenuated the expression of these PANoptosis-related proteins, reversing the observed trends. These findings demonstrated that AU acted as a potent ROS scavenger, providing cytoprotective effects across various PANoptosis pathways.

### 3.4 Studied the anti-PANoptosis effects of AU in mice model of LL-IRI

The role of ROS in acute LL-IRI has been extensively studied, and its overproduction directly leads to increased cellular damage and inflammatory responses. ROS promoted PANoptosis via the activation of multiple cell death pathways, further enlarging tissue damage [46,48]. However, the precise mechanisms by which ROS regulated PANoptosis in LL-IRI remained unclear. H&E staining was first performed to evaluate the biosafety of BFP-H_2_O_2_. As shown in Fig. S12, no obvious histopathological abnormalities were observed in the liver or kidneys of mice injected intravenously with BFP-H_2_O_2_ for 24 h compared with the saline-injection group. The hepatic architecture and renal glomerular and tubular structures remained intact, indicating good biocompatibility of BFP-H_2_O_2_. Next, BFP-H_2_O_2_ was employed to monitor H_2_O_2_ dynamics in mice model of LL-IRI. The mice were divided into three groups: control group (CON), LL-IRI group (bilateral lower limbs subjected to ischemia via orthodontic rubber band ligation at the groin for 3 h, followed by reperfusion for 0.5, 1.0, or 2.0 h), and AU pretreatment group (intraperitoneal injection of 5, 10, 15 mg/kg AU 2 h before modeling) (Fig. 6A). Fig. 6B showed that mice in the CON group had weaker fluorescence signals in the lower limbs, whereas the fluorescence intensity of mice in the LL-IRI group gradually increased with the prolongation of the reperfusion time, indicative of elevated H_2_O_2_ levels. Notably, AU could effectively inhibit the accumulation of H_2_O_2_ during LL-IRI (Fig. 6C).

**Fig. 7:**
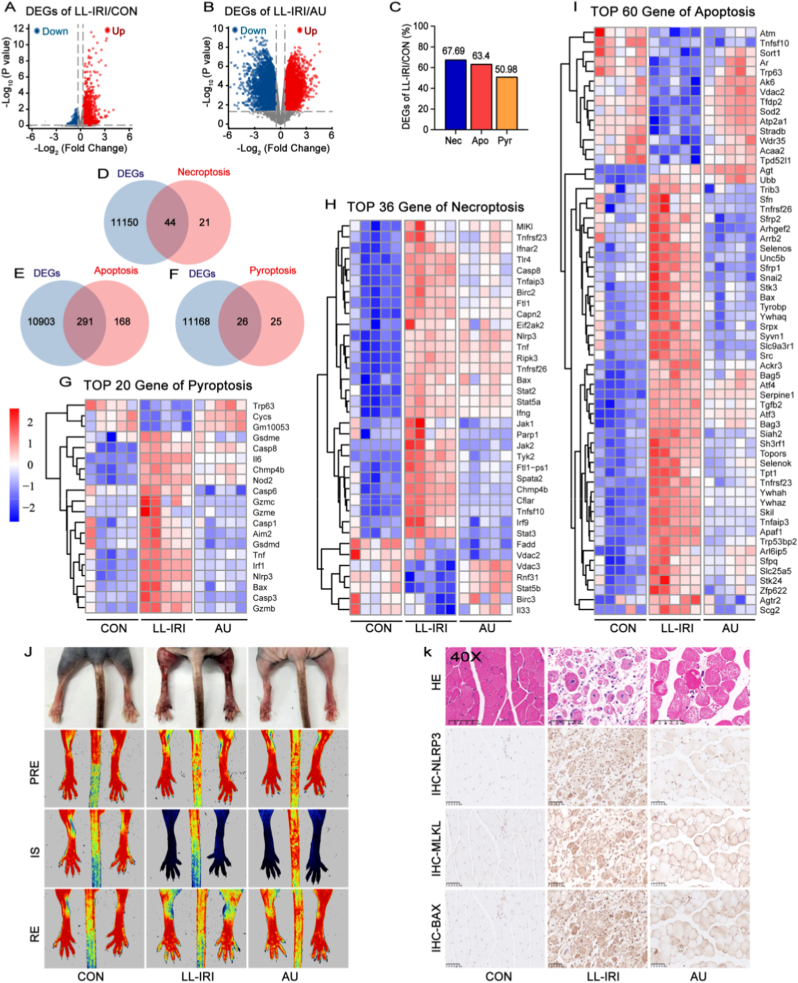
(A) Volcano plot of differentially expressed genes (DEGs) between the LL-IRI and CON groups. Each dot represents one DEG; red: upregulation, blue: downregulation. P value < 0.05&|log_2_FoldChange|>0.5. (B) Volcano plot of DEGs between the LL-IRI and AU groups. P value < 0.05&|log_2_FoldChange|>0.5. (C) Proportions of genes related to pyroptosis, apoptosis, and necroptosis among the identified DEGs. (D, E, F) Venn diagrams show the overlap between DEGs and gene sets of pyroptosis, apoptosis, and necroptosis, respectively. Numbers indicated the count of overlapping genes. (G, H, I) Heatmaps of the top 20, 36, and 60 DEGs involved in pyroptosis, necroptosis, and apoptosis, respectively. Gene expression levels are shown as a gradient from blue (low expression) to red (high expression). Death-related gene sets were obtained from the Gene Ontology (GO) database (http://www.geneontology.org/). n = 5 per group. (J) Representative macroscopic images of the hind limbs from each group, along with laser Doppler perfusion imaging before ischemia (PRE), after 3 h of IS, and 2 h RE. Red indicates blood flow, while blue indicates no blood flow. (K) H&E staining of the gastrocnemius muscle, and IHC analysis of NLRP3, MLKL, and Bax protein expression in the gastrocnemius muscle tissues from each group. n = 5 per group.

To further elucidate the underlying mechanisms, we performed bulk RNA sequencing of gastrocnemius muscle tissues and conducted gene set enrichment analysis (GSEA). Compared with the CON group, LL-IRI remarkably upregulated pyroptosis (50.98 %, 26/51), apoptosis (63.4 %, 291/459), and necroptosis-related genes (67.69 %, 44/65), indicating a predominant role of PANoptosis (Fig. 7A–F). In addition, heatmap analysis demonstrated that AU treatment reversed the expression of PANoptosis-related genes (Fig. 7G–I). Gene Ontology (GO) analysis further revealed that LL-IRI promoted inflammatory and immune responses (BP category) and caused severe mitochondrial damage (CC category), both of which were reversed by AU treatment, aligning with subsequent experimental validation (Figs. S13 and S14). *In vivo* studies also validated these findings. Macroscopic examination showed severe lower limb swelling in LL-IRI mice, which was alleviated by AU treatment. Laser Doppler flow assay showed that during the ischemic stage, there was no blood flow signal in both lower limbs of the LL-IRI group and AU group, while blood flow was restored after reperfusion (Fig. 7J). Histological (H&E) and immunohistochemical (IHC) analyses showed extensive gastrocnemius muscle tissues swelling, cell membrane rupture, nuclear dislocation, and inflammatory infiltration in LL-IRI mice, accompanied by upregulated expressions of NLRP3, MLKL, and BAX. AU treatment markedly attenuated these pathological changes (Fig. 7K).

**Fig. 8:**
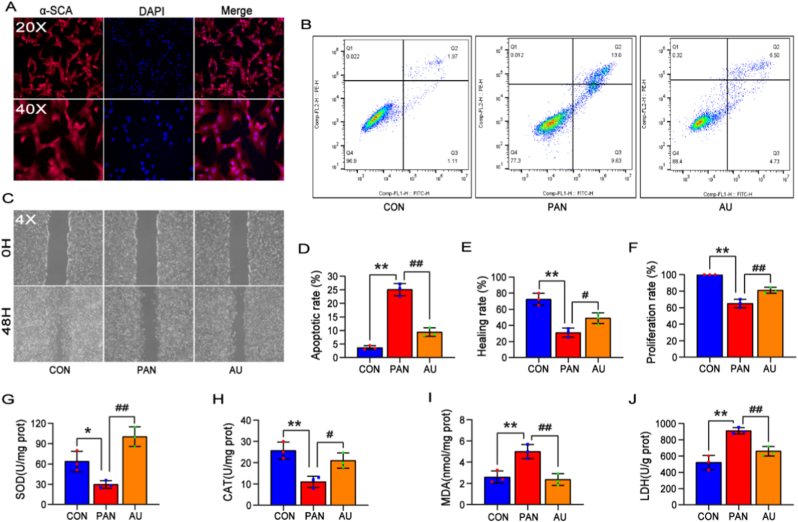
*In vitro* analysis of primary GMCs. (A) Isolation of primary GMCs from mice and identification of α-SCA expression. (B) Apoptosis rates of cells in each group were detected using an apoptosis assay kit. (C) Wound healing assay and quantification of wound closure rates. (D) Quantification of apoptosis rates of cells in each group. (E) Quantification of healing rate. (F) Cell viability in each group was assessed using the CCK-8 assay. (G, H, I, J) Levels of antioxidant enzymes (SOD, CAT) and oxidative stress markers (MDA, LDH) in both cells and culture media were measured using commercial kits. Values are mean ± SD for n = 3. ∗P < 0.05, ∗∗P < 0.01, ^#^P < 0.05, ^##^P < 0.01.

To better align with the transcriptomic analysis, we isolated GMCs from the gastrocnemius muscle tissues of mice and confirmed their identity using α-skeletal muscle actin (α-SCA) immunostaining (Fig. 8A). Using a similar experimental setup as in the L-6 cells model, we conducted flow cytometry-based apoptosis, scratch wound healing, and CCK-8 cell viability assays. Moreover, we quantified key oxidative stress markers, including superoxide dismutase (SOD) and catalase (CAT) as antioxidant enzymes, as well as malondialdehyde (MDA) and lactate dehydrogenase (LDH) as indicators of oxidative stress. As shown in Fig. 8B and D, AU treatment significantly reduced apoptosis rates in the PANoptosis cell model. AU enhanced scratch wound closure (Fig. 8C and E) and improved cell viability (Fig. 8F). Biochemical analysis revealed that AU treatment increased SOD and CAT levels in both cells and culture media (Fig. 8G and H), while reducing MDA and LDH levels (Fig. 8I and J). Collectively, these results illustrated that LL-IRI-induced ROS accumulation exacerbated oxidative stress, triggering inflammatory cascades and PANoptosis. By enhancing endogenous antioxidant enzyme activity and mitigating oxidative stress, AU effectively preserved primary GMCs viability, attenuated PANoptosis, and alleviated LL-IRI.

## 4 Conclusion

In summary, we constructed a novel NIRF probe, BFP-H_2_O_2_, for dynamic monitoring of H_2_O_2_ levels. With the aid of BFP-H_2_O_2_, we not only established a positive correlation between H_2_O_2_ levels and the progression of PANoptosis but also identified AU as the most potent anti-PANoptosis compound among 16 natural products isolated from *PW*. Western blot analysis demonstrated that AU functioned as a cell protector and inhibited the expression of PANoptosis-related proteins, which was consistent with the CLSM imaging results. More importantly, we demonstrated that PANoptosis occurred in GMC of LL-IRI mice for the first time, and AU alleviated LL-IRI by enhancing antioxidant enzyme activity, reducing oxidative stress, and inhibiting PANoptosis. These findings provided a new chemical tool for high-throughput screening of natural anti-PANoptosis drugs, as well as new insights into the mechanisms of PANoptosis and the pathogenesis of LL-IRI.

## CRediT authorship contribution statement

**Tang Deng:** Conceptualization, Data curation, Funding acquisition, Writing – original draft. **Zhanli Peng:** Methodology, Validation. **Jinxi Liang:** Methodology, Validation. **Qinghui Kan:** Formal analysis, Writing – review & editing. **Jin Peng:** Formal analysis, Writing – review & editing. **Zhihao Zhou:** Formal analysis. **Lin Huang:** Formal analysis. **Heng Liu:** Funding acquisition, Methodology, Project administration, Resources, Supervision, Writing – original draft. **Guiyun Jin:** Funding acquisition, Methodology, Project administration, Resources, Supervision, Writing – review & editing. **Chen Yao:** Funding acquisition, Methodology, Project administration, Resources, Supervision, Writing – review & editing.

## Declaration of competing interest

The authors declare that they have no known competing financial interests or personal relationships that could have appeared to influence the work reported in this paper.

## Data Availability

Data will be made available on request.
