# Supplementary material for: Unveiling aucubin-mediated inhibition of PANoptosis in lower limb ischemia-reperfusion injury with a near-infrared H2O2 fluorogenic probe
Source: Mater Today Bio. 2026 Jan 23;37:102845. doi: 10.1016/j.mtbio.2026.102845 (PMC12874344; doi:10.1016/j.mtbio.2026.102845)
Supplement: Multimedia component 1 [file mmc1.docx]

**Supporting Information**

**Unveiling aucubin-mediated inhibition of PANoptosis in lower limb ischemia-reperfusion injury with a near-infrared H_2_O_2_ fluorogenic probe**

Tang Deng^a,b,#^, Zhanli Peng^a,#^, Jinxi Liang^c,#^, Qinghui Kan^a^, Jin Peng^b^, Zhihao Zhou^a^, Lin Huang^a,^, Heng Liu^b,*^, Guiyun Jin^b,*^, Chen Yao^a,*^

^a^ Division of Vascular Surgery, the First Affiliated Hospital, Sun Yat-sen University, Guangzhou 510800, China

^b^ Key Laboratory of Emergency and Trauma of Ministry of Education, Department of Interventional radiology and vascular surgery, The First Affiliated Hospital of Hainan Medical University, Hainan Medical University, Haikou 571199, China

^c^ Department of Ophthalmology, the First Affiliated Hospital of Guangxi Medical University, Guangxi 530021, China

*To whom correspondence should be addressed:

E-mail addresses: [liuheng11b@muhn.edu.cn](mailto:jixueying@muhn.edu.cn) (H. Liu); [13976609625@qq.com](mailto:13976609625@qq.com) (G. Jin); [yaochen@mail.sysu.edu.cn](mailto:yaochen@mail.sysu.edu.cn) (C. Yao).

^#^These two authors contributed equally to this work (T. Deng, Z. L. Peng and J. X. Liang).

**Table of contents**

1. General experimental section
2. Synthesis and characterization of BFP-H_2_O_2_
3. Proposed the reaction mechanism between BFP-H_2_O_2_ and H_2_O_2_
4. Fluorescence data of the designed four NIRF probes BFP-H_2_O_2_
5. The cytotoxicity of BFP-H_2_O_2_ in live cells
6. Fluorescence imaging of endogenous H_2_O_2_
7. Analysis of 16 major components in PW
8. Evaluation of the protective effect of AU on TAK1i/LPS-induced PANoptosis
9. Dose-dependent protective effects of AU on oxidative stress and cell viability in PANoptotic GMCs
10. Biosafety of BFP-H_2_O_2_
11. GO analysis TOP 10 between LL-IRI group and CON group
12. GO analysis TOP 10 between AU group and LL-IRI group

**1. General Experimental Section**

**Materials and instruments:** All chemicals are purchased from commercial suppliers and are not further purified. Unless otherwise stated, all chemicals used in the synthesis are analytical reagent levels. The ultrapure water used in the experiment was prepared by WP-UP-YJ-30 ultrapure water systems. Fluorescence spectra were measured on a Horiba Fluorolog-QM fluorescence spectrophotometer. All pH values were measured with a Sartorius PB-10 pH meter. ^1^H NMR and ^13^C NMR experiments were performed with a JNM-ECZ400S/L1 NMR spectrometer, δ values are in ppm relative to TMS. High resolution mass spectra (HRMS) were measured with an ESI source using a time-of-flight (TOF) detector. Cell imaging experiments were performed using an Olympus FV3000 laser confocal microscope. The cytotoxicity was measured on SepctraMax iD5. The mice imaging experiments were conducted on IVIS Lumina XR small animal optical imaging system.

**Fluorescence Analysis.** The stock solutions of BFP-H_2_O_2_ (1 mM) were prepared in dimethyl sulfoxide (DMSO) and maintained at 4 °C. The fluorescence emission spectra of BFP-H_2_O_2_ were measured in PBS solution containing 30 % ethanol (20 mM, pH 7.4) at 25 ℃. Fluorescence emission spectra were obtained with Xenon lamp and 1.0 cm quartz cells. The fluorescence excitation wavelength was 490 nm with excitation and emission slits of 10 nm, and the emission was collected at 550-750 nm.

**Cell culture and Imaging.** Rat L6 myoblasts (Pricella, China) were maintained in high-glucose DMEM supplemented with 10% FBS and 1% penicillin-streptomycin, with subculturing and cryopreservation performed under standard protocols. Cell migration scratch assay was conducted in 3% FBS/high-glucose medium. Cells (passages <10) were cultured at 37°C in 5% CO₂/5% O₂. H_2_O_2_ levels were imaged using BFP-H_2_O_2_ (10 μM) on an Olympus FV3000 laser confocal microscope with an objective lens (× 60). λ_ex_ = 488 nm, λ_em_ = 600-700 nm.

***In vivo* imaging in LL-IRI model.** Fifteen specific pathogen-free (SPF) male C57BL/6 mice (6-7 weeks old, body weight 25 ± 5 g) were obtained from Hunan Tianqin Biotechnology Co., Ltd. (Hunan, China) and housed in the Animal Experiment Center of Hainan Medical University under controlled conditions (22 ± 1°C, 55% humidity, 12 h/12 h light-dark cycle). All experimental protocols were approved by the Institutional Animal Care and Use Committee (IACUC) of Hainan Medical University.

Mice were randomly divided into three groups. Control (CON) group: received daily intraperitoneal (i.p.) injections of 1 mL normal saline (NS) for 4 days without modeling. LL-IRI group: administered 1 mL NS i.p. daily for 4 days. On day 4, bilateral hindlimb ischemia was induced by applying orthodontic rubber bands (3.5 oz, 1/8" width, 3.18 mm; 3M Unitek) to the femoral grooves for 3 h, followed by 2 h of reperfusion. AU pretreatment group: received 5, 10, 15 mg/kg AU (optimal dose determined by probe-based screening) in 1 mL NS via i.p. injection daily for 3 days. On the fourth day, 5, 10, and 15 mg/kg of AU were administered via intraperitoneal injection 2 h before establishing the LL-IRI model, with the modeling method identical to the LL-IRI group. All mice received BFP-H_2_O_2_ (250 μM, 200 μL) via in stiu injection. Following anesthesia, *in vivo* fluorescence imaging was performed using IVIS Lumina XR small animal optical imaging system. The imaging parameters were set with an excitation wavelength of 520 nm, and fluorescence emission was recorded at 650 nm.

Mice were euthanized by cervical dislocation under deep anesthesia (pentobarbital, 50 mg/kg i.p.) immediately post-modeling. Bilateral gastrocnemius muscles were collected for: Bulk RNA sequencing (Illumina NovaSeq 6000); Western blotting (WB) analysis; Hematoxylin-eosin (HE) staining; Immunohistochemistry (IHC).

**PW extract preparation and intervention.** Fresh PW samples collected from Danzhou, Hainan Province were ground into powder using liquid nitrogen. Briefly, 2 g aliquots were weighed into 15 mL centrifuge tubes, mixed with 20 mL methanol, and homogenized by vertexing. After 30-min ultrasonic extraction (40 kHz, 25°C), 1 mL aliquots were centrifuged (12,000 ×g, 4°C, 10 min), filtered through 0.45 μm membranes, and analyzed by HPLC (Agilent 1260 Infinity II) under optimized chromatographic conditions

**Isolation and validation of primary cells.** Primary gastrocnemius muscle cells (GMCs) were isolated from C57 suckling mice via enzymatic digestion (collagenase/dispase, Procell, China) and validated by α-sarcomeric actin (α-SCA) immunofluorescence. Cells were cultured in high-glucose DMEM (Gibco) supplemented with 10% FBS (ExCell Bio) and 1% penicillin-streptomycin (Beyotime), routinely subcultured and cryopreserved. Experiments used cells below passage 5 (37°C, 5% CO₂/5% O₂).

**Cell migration scratch assay.** Confluent GMCs monolayers in 6-well plates were vertically scratched using 200 μL pipette tips. Wound closure rates at 0 h and 48 h were quantified via phase-contrast microscopy and analyzed with ImageJ software (MRI Wound Healing Tool plugin).

**Detection of cell proliferation and apoptosis.** GMCs was inoculated into 96-well plates, CCK-8 reagent was added, and the cell proliferation rate was calculated according to the OD value. GMCs was inoculated into a 6-well plate, GMCs was digested with pancreatic enzyme without EDTA, centrifuged, the supernatant was removed, the cell stock solution was diluted to 3×10^6^ PCS /ml, 6 multiple tubes/groups, 100μl/tube, and the supernatant was removed by centrifugation again. The 1×Binding Buffer 100μl re-suspension cells were added, and PI Staining Solution 10 µl and Annexin V-FITC 5µl light staining reaction were added, and then the staining was thoroughly mixed with 1×Binding Buffer 400 µl. The apoptosis rate of each group was detected by flow cytometry.

**Analysis of Antioxidant and Oxidative Stress Markers in GMCs Lysate Supernatants.** Levels of superoxide dismutase (SOD), catalase (CAT), lactate dehydrogenase (LDH), and malondialdehyde (MDA) in cell culture supernatants were quantified using colorimetric assays (SOD, CAT, MDA) and microplate-based methods (LDH), following the manufacturer’s protocols (Nanjing Jiancheng Bioengineering Institute, China).

**H&E staining of Gastrocnemius muscle.** The Gastrocnemius muscle tissues of mice were fixed with 4% formaldehyde solution, embedded in paraffin after dehydration, sliced in paraffin, and then stained by HE after dehydration and rehydration.

**Western Blot.** Western blot analysis was performed on protein lysates extracted from L6 cells. Protein extraction was performed using RIPA lysis buffer (Bode Bioengineering, Wuhan, China). Following SDS-PAGE separation, proteins were transferred onto PVDF membranes and blocked with 5% skim milk for 90 min at room temperature. Membranes were incubated overnight at 4°C with primary antibodies in a refrigerated shaker, followed by 1 h incubation with HRP-conjugated secondary antibodies at room temperature. Protein bands were visualized using chemiluminescence detection after thorough washing. Band intensity was quantified using ImageJ software with GAPDH as loading control. Antibody specifications: p-MLKL (Bioss, China): Rabbit, 1:1000；Other antibodies (Proteintech, China): GSDMD (Rabbit) 1:2000；CASP1 (Rabbit) 1:2000；NLRP3 (Rabbit) 1:2500；MLKL (Rabbit) 1:10000；Bcl-2 (Rabbit) 1:1500；CASP3 (Mouse) 1:2000；Bax (Rabbit) 1:8000；GAPDH (Mouse) 1:5000

**Immunohistochemistry.** Gastrocnemius muscle sections were deparaffinized, rehydrated, and subjected to antigen retrieval with citric acid buffer. Endogenous peroxidase activity was blocked with 3% H_2_O_2_. After PBS rinsing, sections were blocked with 4% goat serum and incubated with primary antibodies at room temperature. DAB staining was performed followed by hematoxylin counterstaining, graded ethanol dehydration, xylene clearing, and mounting. Antibody dilutions: NLRP3 (Rabbit) 1:800；MLKL (Rabbit) 1:1000; Bax (Rabbit) 1:2000.

**Laser doppler imaging of hindlimb ischemia.** Hindlimb perfusion was assessed using a PeriScan PIM3 laser Doppler system (Perimed AB, Sweden) at baseline (5 min pre-ischemia), 3 h post-ischemia, and 2 h post-reperfusion. Mice were anesthetized with 1.5% isoflurane and positioned prone on a 37°C thermostatic blanket to minimize thermal fluctuations. Blood flow in ischemic and contralateral limbs was quantified by mean pixel intensity within regions of interest. Perfusion values were visualized as color-coded maps (red: maximal perfusion; dark blue: minimal perfusion).

**Statistical analysis of data.** Results are expressed as mean ± standard deviation (x ± SD). The sample mean was compared pairwise by one-way ANOVA. P<0.05 was considered statistically significant. All data were statistically analyzed by GraphPad software.

**2. Synthesis and characterization of BFP-H_2_O_2_**


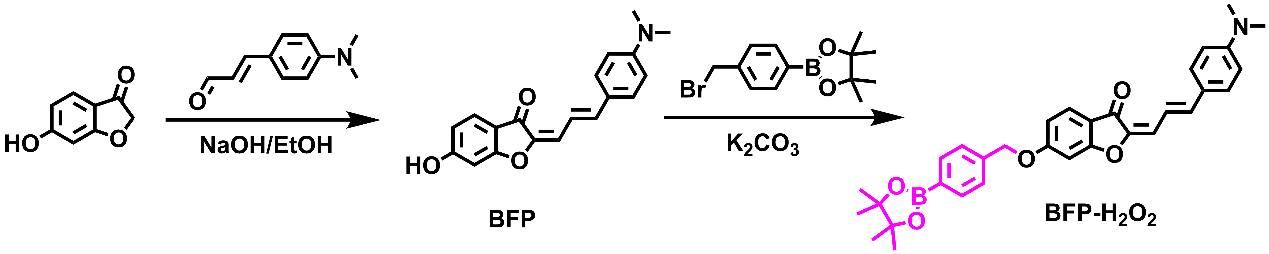


**Synthesis of (E)-2-((E)-3-(4-(dimethylamino)phenyl)allylidene)-6-((4-(4,4,5,5-tet ramethyl-1,3,2-dioxaborolan-2-yl)benzyl)oxy)benzofuran-3(2H)-one (BFP-H_2_O_2_)**

BFP (307 mg, 1.0 mmol), potassium carbonate (276 mg, 2.0 mmol), and 2-(4-(bromomethyl) phenyl)-4,4,5,5-tetramethyl-1,3,2-dioxaborolane (445 mg, 1.5 mmol) were sequentially added to a solution of N, N-dimethylformamide (10.0 mL). The reaction mixture was heated to 80 °C and stirred for 6 h. The progress of the reaction was monitored by TLC. Upon completion, the solvent was removed under reduced pressure. The crude product was redissolved in dichloromethane and purified by silica gel column chromatography using dichloromethane as the eluent to afford BFP-H_2_O_2_ (183 mg, 35 %). ^1^H NMR (400 MHz, CDCl_3_): 7.85 (d, *J* = 8.0 Hz, 2H), 7.67 (d, *J* = 8.6 Hz, 1H), 7.44 (d, *J* = 8.1 Hz, 4H), 7.07 (dd, *J* = 15.4, 11.5 Hz, 1H), 6.92 (d, *J* = 15.5 Hz, 1H), 6.80-6.77 (m, 2H), 6.74 (d, *J* = 1.6 Hz, 1H), 6.67 (d, *J* = 8.8 Hz, 2H), 5.18 (s, 2H), 3.02 (s, 6H), 1.35 (s, 12H); ^13^C NMR (100 MHz, CDCl_3_): 181.72, 167.11, 165.75, 151.06, 147.12, 141.93, 138.76, 135.17, 131.01, 129.02, 126.53, 125.46, 124.56, 116.42, 116.05, 115.32, 112.23, 111.98, 97.38, 83.89, 70.51, 40.18, 24.84; HRMS m/z: C_32_H_34_BNO_5_ [M + H]^+^ calcd for 524.2608 found 524.2609.


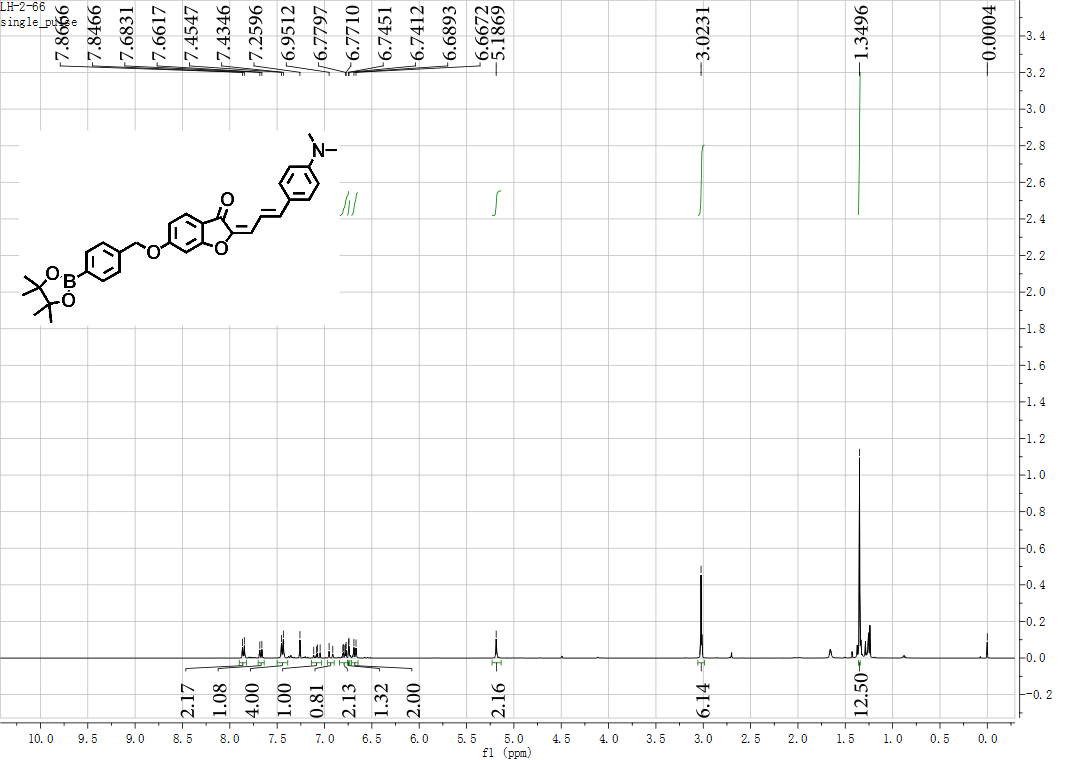


**Figure S1.** ^1^H NMR (400 MHz) spectra of BFP-H_2_O_2_ in CDCl_3_.


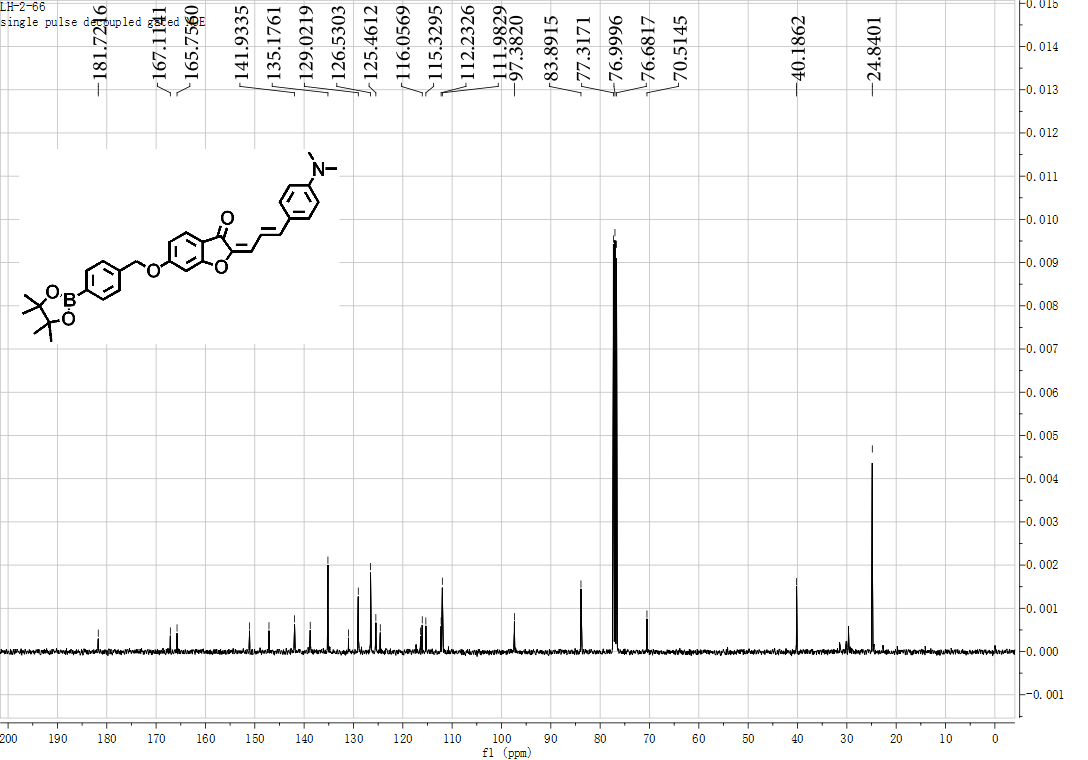


**Figure S2.** ^13^C NMR (101 MHz) spectra of BFP-H_2_O_2_ in CDCl_3_.


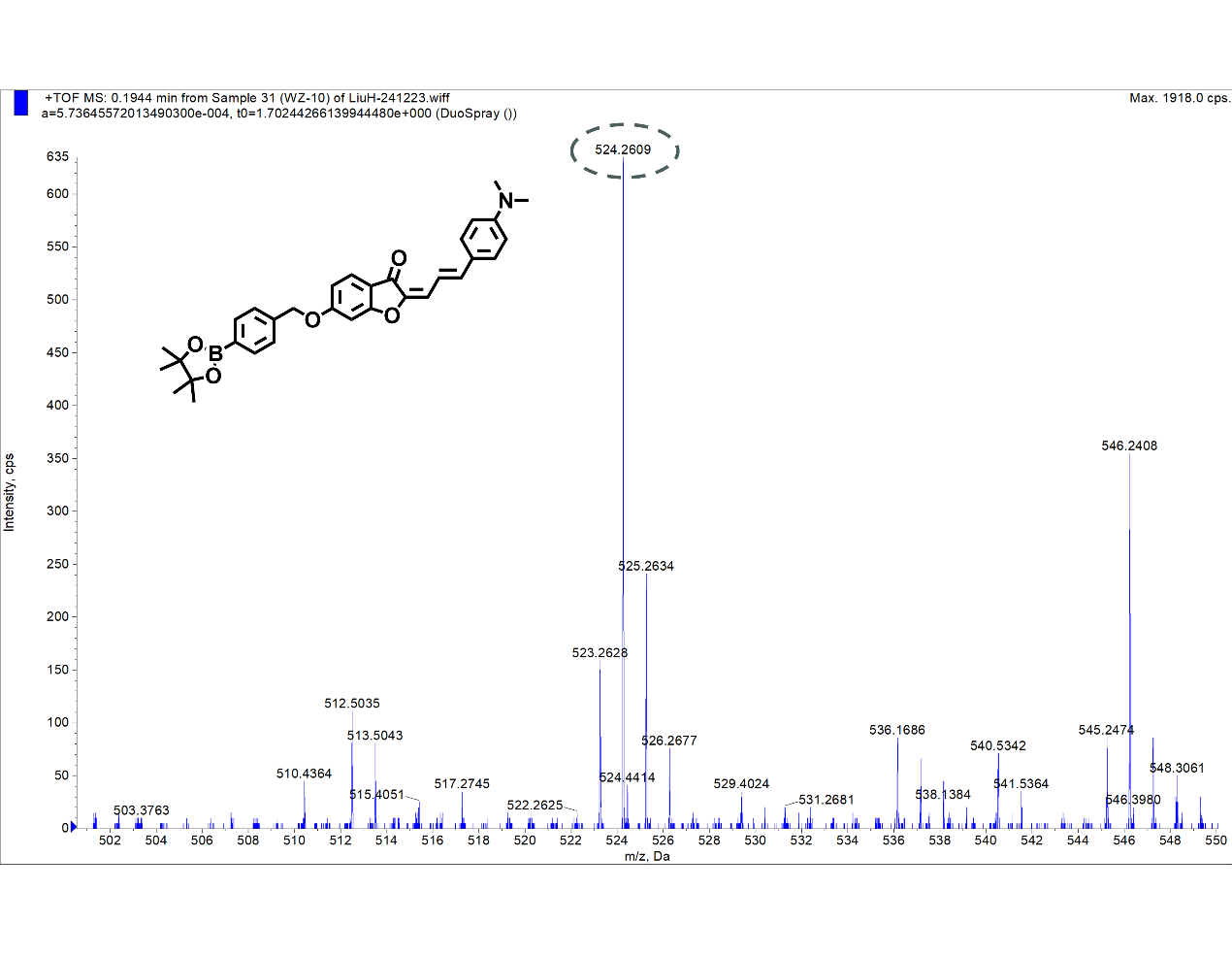


**Figure S3.** HRMS spectra of BFP-H_2_O.

**3. Proposed the reaction mechanism between BFP-H_2_O_2_ and H_2_O_2_**

HRMS experiments were conducted to gain further insight into the reaction mechanism of BFP-H_2_O_2_ and H_2_O_2_. HRMS results showed the presence of a major peak at m/z 330.3350 assigned to the complex BFP ([M + Na]^+^), which was different from the mass peak of BFP-H_2_O_2_ at m/z = 524.2609 ([M + H]^+^).


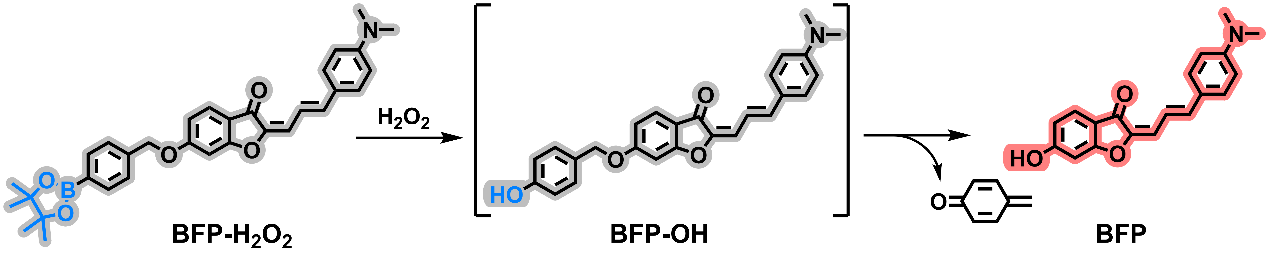


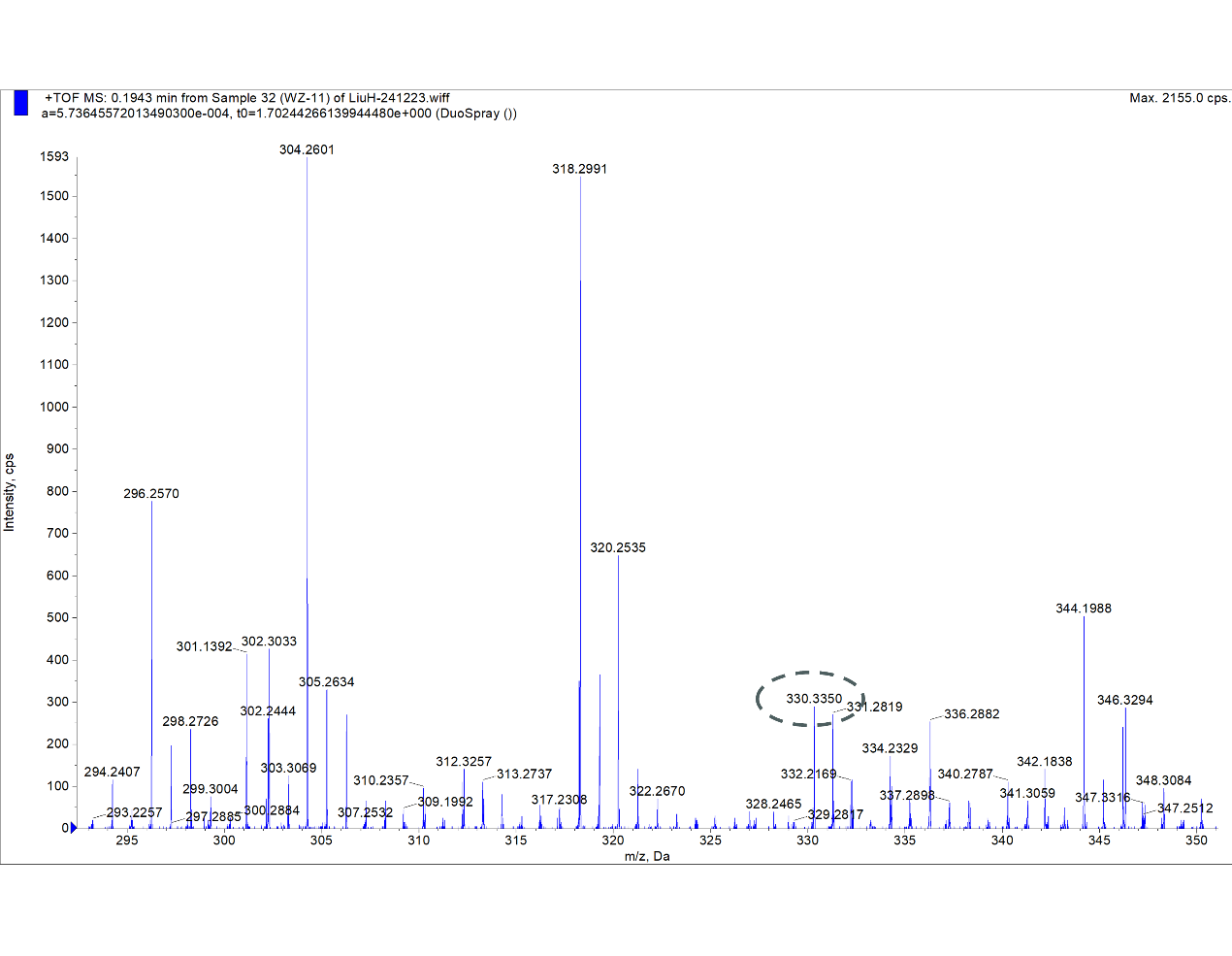


**Figure S4.** HRMS spectra of the reaction product between BFP-H_2_O_2_ and H_2_O_2_.

**4. Fluorescence data of the designed four NIRF probes BFP-H_2_O_2_**


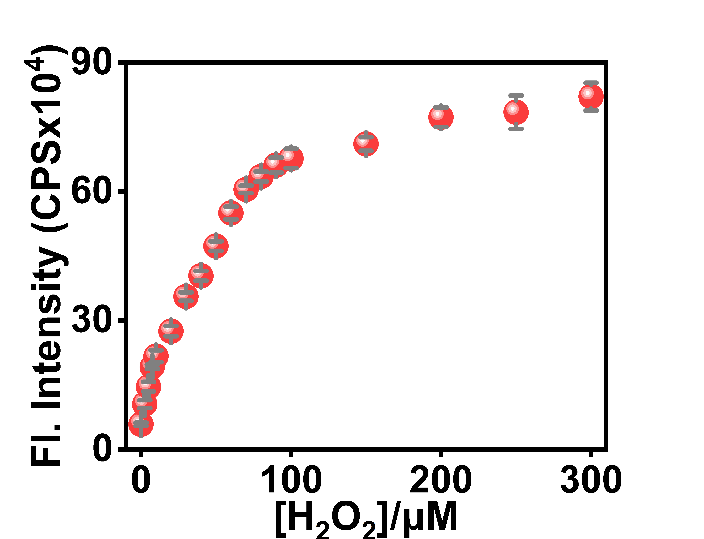


**Figure S5.** Fluorescence intensity at 650 nm of BFP-H_2_O_2_ (10 μM) vs 0-300 μM H_2_O_2_. λ_ex_ = 490 nm.


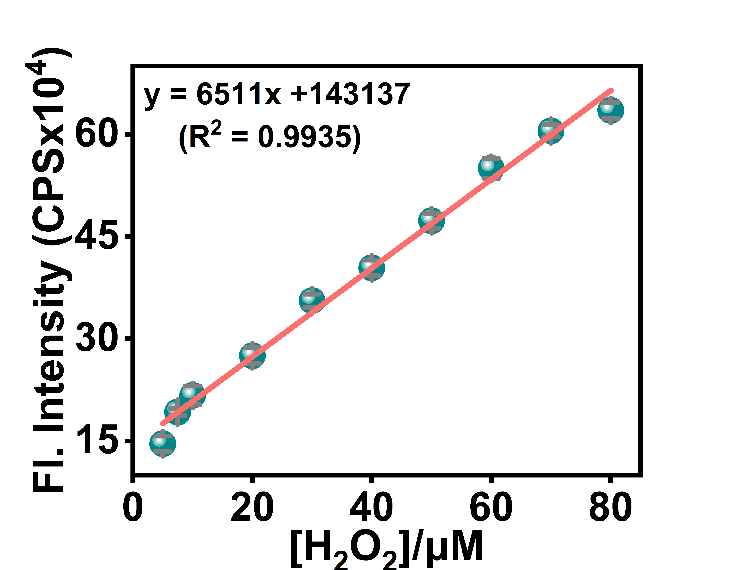


**Figure S6.** The linear curves of fluorescent intensity at 650 nm of BFP-H_2_O_2_ (10 μM) against various H_2_O_2_ concentrations. λ_ex_ = 490 nm.

**5. The cytotoxicity of BFP-H_2_O_2_ in live cells**


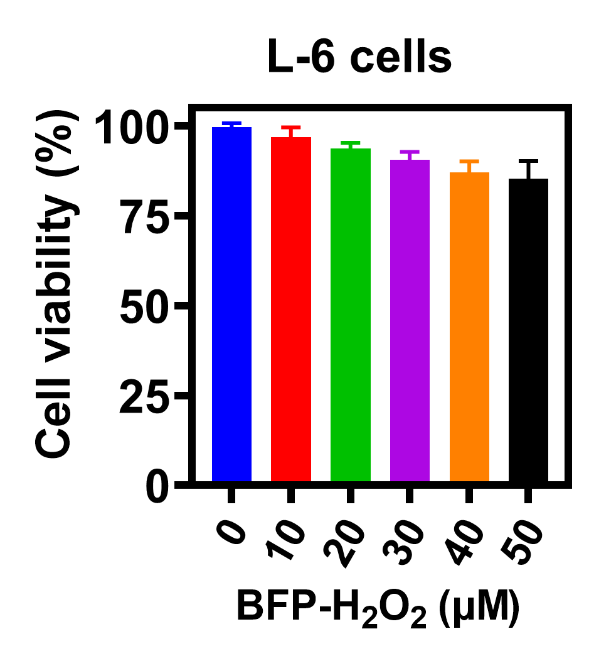


**Figure S7.** Cell viability of L-6 cells in various concentrations of BFP-H_2_O_2_ for 24 h was determined by CCK-8 assay. The experiments were repeated three times, and the data were shown as mean (± S.D.).

**6. Fluorescence imaging of endogenous H_2_O_2_**


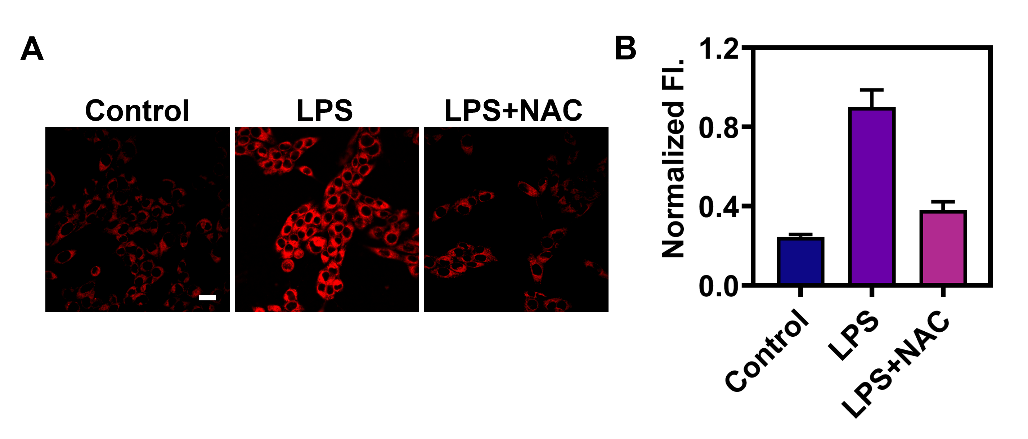


**Figure S8.** Images of L6 cells incubated with BFP-H_2_O_2_ (10 μM) for 30 min as control; stimulated with LPS (20 μg/mL) for 12 h, and then incubated with BFP-H_2_O_2_ (10 μM) for 30 min; stimulated with LPS (20 μg/mL) for 12 h, treated with NAC (1 mM) for 30 min, and subsequently incubated with BFP-H_2_O_2_ (10 μM) for 30 min. Scale bar: 20 μm. Values are mean ± SD for n =3.

**7. Analysis of 16 major components in PW**


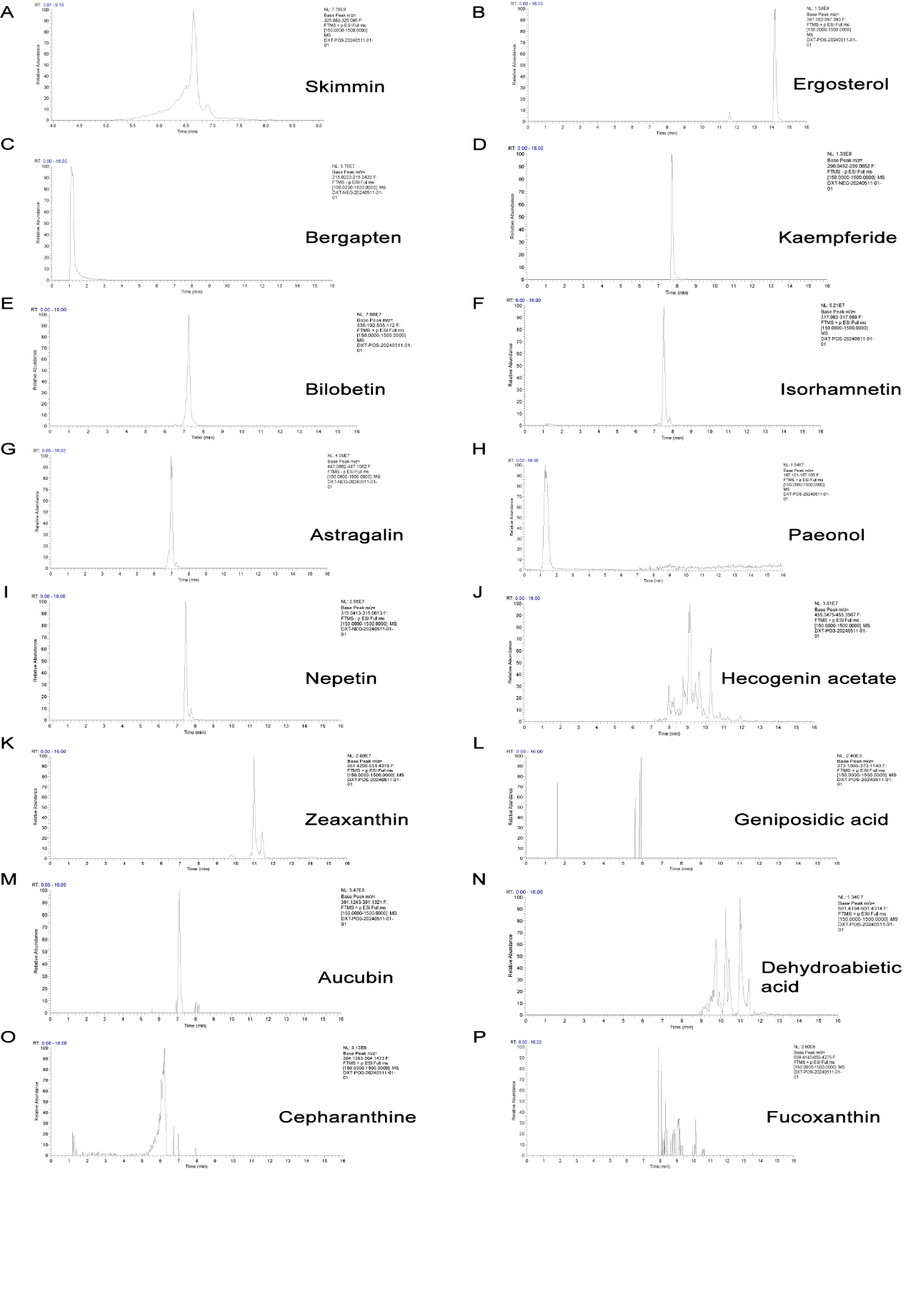


**Figure S9.** Analysis of 16 major components in PW using high-performance liquid chromatography.

**8. Evaluation of the protective effect of AU on TAK1i/LPS-induced PANoptosis**


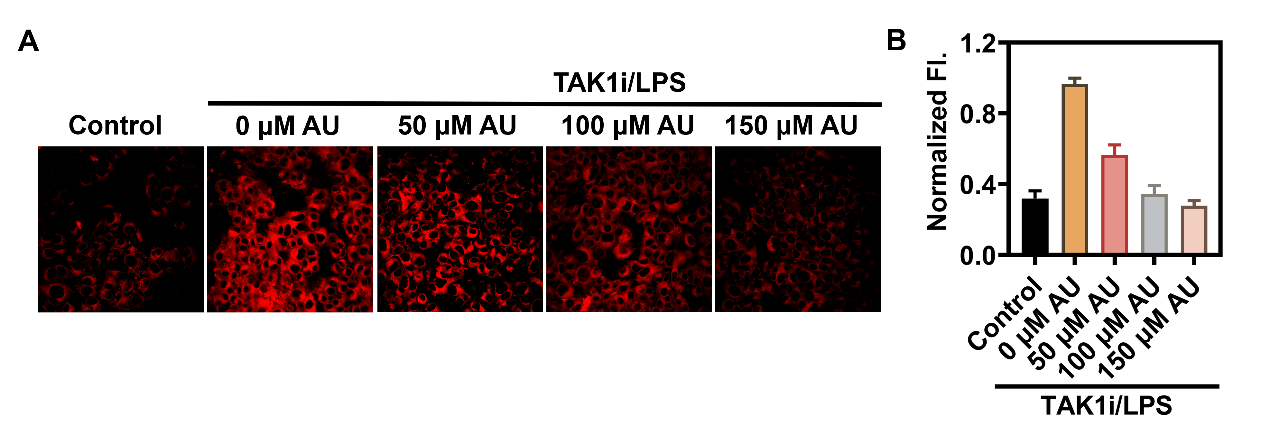


**Figure S10.** (A) Images of L6 cells pretreated with various concentrations of AU (0, 50, 100, 150 μM), then stimulated with **TAK1i (0.1** μM**) for 1 h** and **LPS (0.1** μg/mL**) for another 3 h, followed by staining with** BFP-H_2_O_2_ (10 μM) for 30 min.

**9. Dose-dependent protective effects of AU on oxidative stress and cell viability in PANoptotic GMCs**


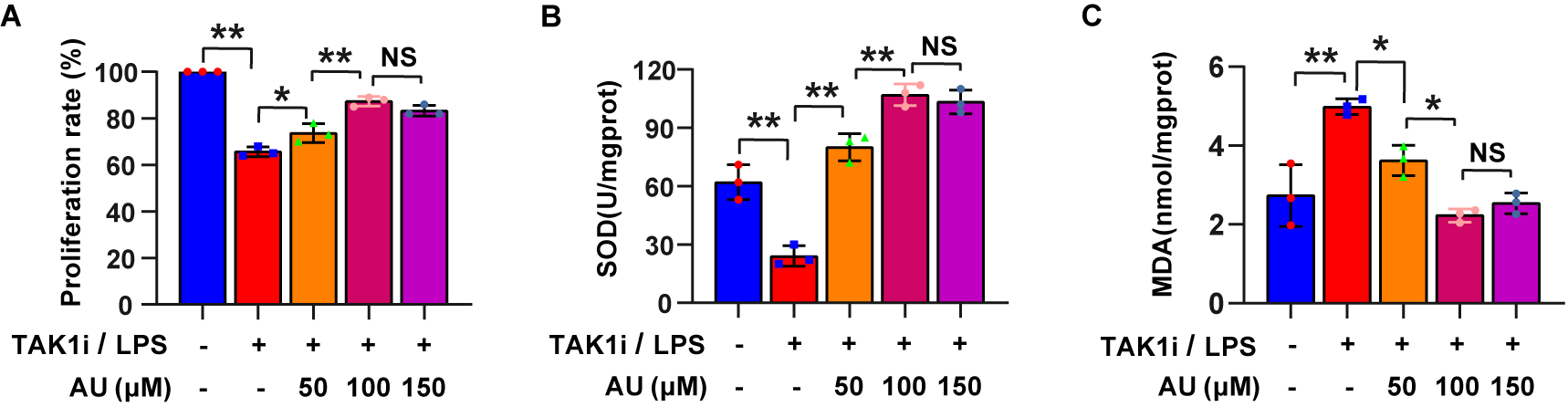


**Figure S11.** AU alleviated oxidative stress in a dose-dependent manner and enhanced cell viability and antioxidant enzyme levels in PANoptotic GMCs.
(A) Relative cell viability of TAK1i/LPS-induced PANoptotic GMCs treated with different concentrations of AU, as determined by the CCK-8 assay.
(B-C) Levels of the antioxidant enzyme superoxide dismutase (SOD) and the oxidative stress marker malondialdehyde (MDA) in cells and culture medium, measured using commercial assay kits. Data are presented as mean ± SD (n = 3). *P < 0.05, **P < 0.01.

**10. Biosafety of BFP-H_2_O_2_**


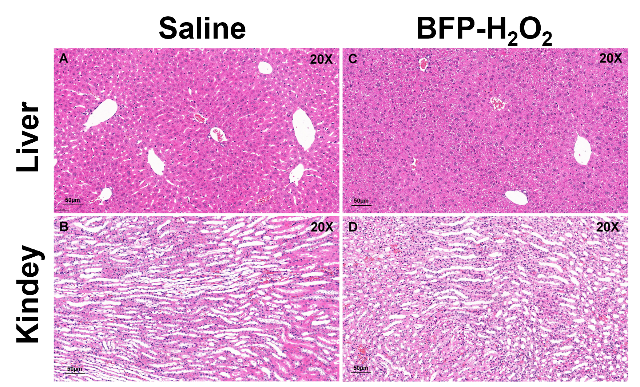


**Figure S12.** H&E staining of major organs (liver, kidney) of mice injected intravenously with saline or BFP-H_2_O_2_ (250 μM, 200 μL) for 24 h. Scale bar: 50 μm.

**11. GO analysis TOP 10 between LL-IRI group and CON group**


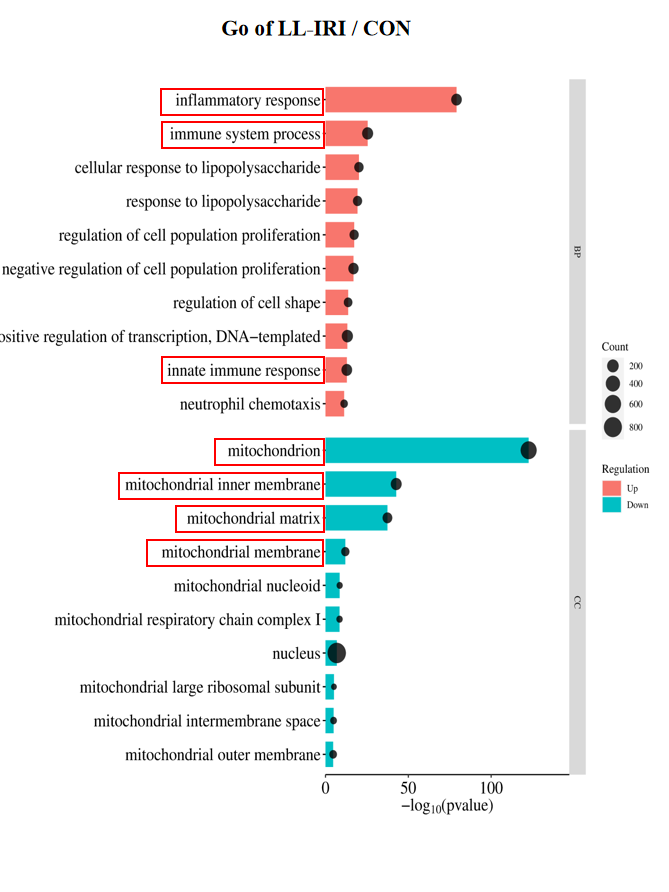


**Figure S13.** GO analysis TOP 10 between LL-IRI group and CON group. Red bars indicate the top 10 upregulated biological processes (BP) in the LL-IRI group compared to the CON group, while blue bars indicate the top 10 downregulated cellular components (CC). BP: Biological Process; CC: Cellular Component. Key terms include inflammatory response, immune system process, innate immune response, and mitochondrial structures (mitochondrion, mitochondrial inner membrane, mitochondrial matrix, mitochondrial membrane). n = 5.

**12. GO analysis TOP 10 between AU group and LL-IRI group**


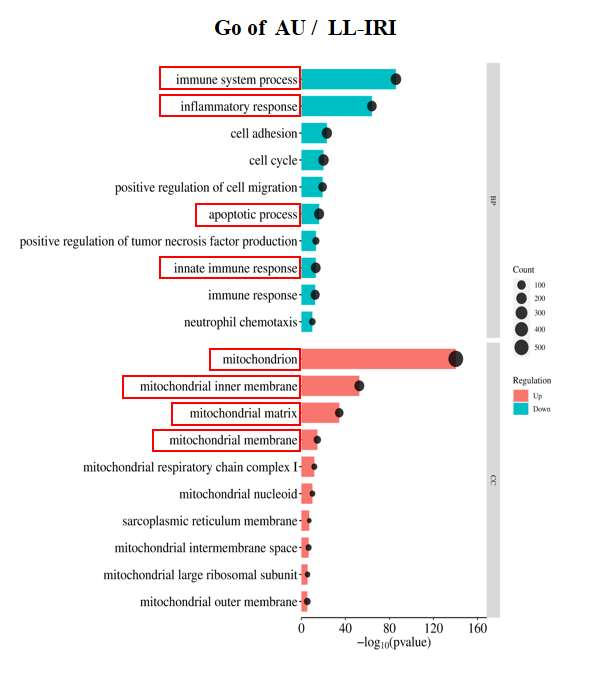


**Figure S14.** GO analysis TOP 10 between AU group and LL-IRI group. Blue bars indicate the top 10 downregulated biological processes (BP) in the AU group compared to the acute LL-IRI group, while red bars indicate the top 10 upregulated cellular components (CC). BP: Biological Process; CC: Cellular Component. Key terms include inflammatory response, immune system process, innate immune response, and mitochondrial structures (mitochondrion, mitochondrial inner membrane, mitochondrial matrix, mitochondrial membrane). n = 5.
